# The Report of the English Commission on the Pasteur Method

**Published:** 1887-10

**Authors:** E. C. Spitzka


### EDITORIAL DEPARTMENT.

### THE REPORT OF THE ENGLISH COMMISSION ON THE PASTEUR METHOD.

A CRITICISM. BY E. C. SPITZKA, M. D.

In its issue of July 2d, the *British Medical Journal* editorially exclaims that the report of the British Hydrophobia Commission, while "not a lengthy document, in a comparatively few paragraphs affords the most complete and powerful defense of M. Pasteur's method, and the most crushing reply to his critics yet published." We confess that our curiosity was aroused by this announcement. Truly it must be as able and thorough as it is a laconic report which could vindicate M. Pasteur and crush his critics satisfactorily, for in this M. Pasteur signally failed himself, as we shall show hereinafter. We have thoroughly analyzed the report to which the *British Medical Journal* refers, and regret to be compelled to admit that it is precisely as unsatisfactory and unconvincing as M. Pasteur's notoriously inconsistent pronunciamentos. Indeed, it is not necessary to go beyond the editorial in question to show this. We reproduce the following, *verbatim :*

"The report also deals with the question raised mainly by v. Frisch in Vienna and Lutand in Paris, whether the inoculations of Pasteur may not themselves be responsible for the deaths of some of his patients. The Committee is of opinion that there is no evidence that any deaths have been caused by the first or ordinary method, but that since the "intensive" method has been used deaths have occurred under conditions which have suggested that they *were due to the inoculations* rather than to the infection from the rabid animal. The case of the man Goffi, an attendant at the Brown Institute, who was bitten on September 4th, was placed under M. Pasteur's care on the following day, but died on October 19th from acute ascending (motor) paralysis in St. Thomas' Hospital, *rather tends to confirm this fear, for Mr. Horsley has found that he died of hydrophobia.* The report favors the assumption that Goffi *died of the virus of the cat by which he was bitten,* chiefly on the ground that the period of incubation was longer than would have followed the inoculation of virus of the highest intensity. This statement, however, appears to go near to begging the whole question, for the matter in dispute is the behavior of the "intensive virus" in man, and a most important element is the duration of incubation. The unusual nature of the symptoms also requires explanation, for the suggestion that the cases hitherto described under the term "acute ascending paralysis," or Landry's paralysis, are in many instances examples of the dumb or paralytic form of rabies in man, rests upon this single observation of Mr. Horsley's, and ignores the fact that a large proportion of such cases recover. That M. Pasteur himself shares to some extent the apprehensions which have been expressed is shown by the fact that he has already modified his intensive method; instead of using a whole series of fourteen cords, from fourteen days to one day old, during the short period of seven days, followed by six other injections on the four following days, again ending with a cord only dried for one day, he over-spreads the injections over fifteen days, and the most virulent cord used has been dried for five days."

We do most respectfully submit, that neither the British Hydrophobia Commission nor the *British Medical Journal's* editorial staff are clear in their own minds, either as to what rabies is, what Pasteur has done, *or what their own words mean!* It is a striking illustration of what we have seen elsewhere adverted to that in no department of thought are medical observers so deficient as in logic. Let us study the alternative presented, leaving out of consideration the fact that the report is self-contradictory as to the cause of the death of Goffi. If it were true that Goffi died from the bite of a cat, as the British Hydrophobia commission claims, would that not be the most positive condemnation of Pasteur's procedure, demonstrating *the utter valuelessness* of his intensive method? Goffi was inoculated by this intensive method under the most favorable auspices, the day after he was bitten. He died. If the intensive method could not save his life—if Pasteur acknowledges that his milder method was not sufficiently protective, what value are we to attribute to his intermediate method?

We refer to this branch of the report in order to show our readers on what hollow presumptions and careless theorizing that minority of English physicians who sustain Pasteur, base their conclusions. The commission includes the names of many prominent scientists, among them the distinguished veterinarian Fleming. But it does not appear that the slightest attempt was made to analyze and weigh the clinical evidences of real or spurious rabies in the animals experimented on. The whole matter seems to have been left in the hands of Mr. Victor Horsley, the Secretary, whose ability to discriminate in any matter involving the use of judgment and well-balanced reasoning, we need no other comment on, than the *British Medical Journal's* admission that he cannot distinguish between Landry's paralysis—a well-recognized and recoverable form of nervous disease in man—and rabies.

We should cover this humiliating admission regarding Mr. Horsley, with the mantle of charitable silence, but unfortunately he has shown what in American politics is called "offensive partisanship," in his letters in the *British Medical Journal*, too prominently to deserve much mercy at the hands of others. When a respectful request was made of him to answer certain questions relating to the symptoms of rabbit rabies, he sneeringly referred the questioner to M. Pasteur, and dictatorially declared that no one had a right to question M. Pasteur's results.

It will thus be seen that while the report of the Hydrophobia Commission is calculated to foster the Pasteur agitation and to keep up the rabies craze, it cannot, even after a very superficial perusal weigh as a feather in the balance in favor of the enthusiastic would-be Jenner of Paris. Other competent, and it seems to us more careful and thorough, investigators have come to very different conclusions. We cite the following from the *Medical and Surgical Reporter* of July 9th.

> The Belgian Government, in consequence of requests made in the Chamber of Deputies, lately deputed three Belgian physicians residing in Paris—Drs. Grandjeau, DeBrun and Peeters—to investigate and report on M. Pasteur's method of preventive treatment of hydrophobia, and to decide upon the advisability of founding a Pasteur Institute in Belgium. The report of these gentlemen, as the Chamber has just been informed, is decidedly averse to such a step. It was decided by the reporters that Pasteur's method is not as yet sufficiently established, and one of them, Dr. De Brun, doubted whether there was any efficacy at all in the treatment. In this last view, Van der Corput, 'Belgium's chief specialist,' coincides."

The Austrian Government has withdrawn its subsidy to the Pasteur Institute, and the Emperor of Austria, to plaster up this wound to Pasteur's vanity, has created him a Baron!

No one who has followed up the rapid changes of opinion and practice indulged in by Pasteur, involving at least six different propositions as to the length of time his virus is protective for, and no less than three different "potencies" of virus, but will admit that his experiments with human beings were altogether premature. Indeed Frisch, who, as long as he expressed himself favorably regarding Pasteur's researches, was lauded to the skies by the latter and as violently attacked as soon as he ventured to differ from him, was enabled to practically predict the deaths which followed from the use of the intensive virus, for he found singularly untoward results in dogs. In his attack on Frisch, Pasteur makes the singular admission that the virus Frisch used—although obtained from Pasteur directly—may have changed in character! We think it may have done so; we are quite certain that M. Pasteur's virus has also changed in character—that is, so far as a thing whose origin, nature and destiny are altogether problematical, may be regarded as being a vacillating and uncertain quantity.

It is exceedingly unfortunate that M. Pasteur is so frightfully inaccurate in his citation of what he regards evidence proving him to be a modern Jenner. The great surgeon and pathologist, Billroth, in endorsing Frisch's experimental conclusions, ventured to pronounce Pasteur's protective authrax inoculations a *fiasco.* As Pasteur's reputation on this score rests almost exclusively on the claim which he has loudly urged—that anthrax has been practically exterminated in France by him—he was touched to the quick by this, and in reply said that the Berlin Laboratory, although originally reserved as to the value of the anthrax inoculations, had more recently, after a repeated test of the matter, endorsed him. Frisch made special inquiries to see if this were true,* and found that

* Discussions in the Vienna Medical Society of June 11th, 1887. Deutsche medizinische wochenschrift, 1887, p. 559.

it was not! The last declaration emanating from that laboratory, based on researches of Koch, Gaffky and Laffler, regarded the anthrax inoculations as of doubtful value. And in digging up the records Frisch came across a statement emanating from the same parliament, whose Hydrophobia commission has now endorsed Pasteur, which is not at all calculated to increase Pasteur's prestige* To those interested in the matter we would urge the perusal of Frisch's reply; it is replete with evidence that Pasteur, unable to meet his adversary squarely on the basis of facts, resorts to evasion and adroit quibbling in attempting to keep up the appearance of a scientific discussion. The latest official statement from Koch of Berlin proves that, to put it mildly, Pasteur relies on his imagination for his facts.

Meanwhile, news comes to us of the forty-fifth death after inoculation † in Pasteur's laboratory. Lord Doneraile though bitten through a glove by a vixen and promptly treated by Pasteur, is also dead, while his coachman, bitten by the same animal on the unprotected hand, is alive and well.

To us, on this side of the Atlantic, it seems that one good will come out of this discussion. We are in a fair way to find out what is rabies in the dog. As soon as that question is clinically settled, we will ascertain what is rabies in man. Most of the evidence we are in receipt of is of the negative kind, showing what is *not* rabies in man, the horse and the dog. Of this kind of evidence we append a few samples.

"At Hunter's Point yesterday a report that Otto Nunbeck, aged 9 years, of 114 East Fourth Street, is suffering from hydrophobia, was denied. The physician attending him said the boy was simply suffering from malaria. He was bitten on the upper lip by a neighbor's dog, and though the animal was not and is not mad, the wound was promptly cauterized. The lad suffers considerable pain, and his outcries caused some gossips in the neighborhood to spread the report that the case was one of hydrophobia." ‡

---

* An enzootic of Anthrax having broken out in Cheshire, Mr. Gardner, in the Commons, interpellated Lord Manners as to the advisability of introducing Pasteur's protective inoculations in England. The reply was that the control experiments made in England had not furnished sufficiently reliable results to justify its introduction.

† A Spaniard named Ramon, bitten by a wolf on the fifteenth of February. The report does not state whether this was not also, like one of the Russian cases, an instance of bad surgery.

‡ N. Y. *Times*, July 29th, 1887.

The same paper contained the following announcement on March 12th :

Charles Berg, station master at Winfield Station, Long Island, died on Jan. 18 in St. Catherine's Hospital at Williamsburg. Berg was attended by Dr. Combes, of Newtown, who pronounced him to be suffering from hydrophobia, but the physicians at the hospital gave meningitis as the cause of death. Berg was bitten by a dog some months before he died. After the post-mortem examination Dr. Combes still held that it was a case of hydrophobia, while the physicians of the hospital contended that it was not. The case excited considerable interest among the medical fraternity, and some doctors, on learning a history of the case, sided with Dr. Combes. The latter took the spinal cord and brain to Prof. Ira Van Giesen, of the New York College of Physicians and Surgeons, for microscopic examination. Prof. Van Giesen has given in writing the result of the examination, which sustains Dr. Combe's diagnosis.

We wrote to the hospital physicians for a history of the case and received a reply embodying the following, which will possibly indicate to our readers why we suppressed our curiosity as to the new discovery which enabled Professor Van Giesen, if correctly reported, to decide the case to be one of Hydrophobia :

"Patient was admitted to this hospital as a supposed case of hydrophobia, having been bitten by a dog two years previous. On examination patient was found to be in a very excited condition. Temperature and pulse normal, refused to answer questions when spoken to, would not take food or drink. Towards evening patient became violently delirious. Temperature rapidly rose to 103°, face very much flushed and pupils widely dilated. Diagnosis of acute meningitis was made and confirmed by one of the visiting physicians. Patient died the following morning at 4 A. M.

*Autopsy* * * * * *Brain* congested, fibrin along the vessels, cloudiness in the arachnoid * * * * * *. AUGUST HOERLE,

*House Surgeon.*

A very interesting account is also furnished by Dr. Harbaugh, V. S., of Richmond, Virginia,* concerning the case of Mr. Garnett. This gentleman had been bitten by a mare suffering from œstro-mania. We quote from Dr. Harbaugh's statement :

Mr. G. informed me that one gentleman drove through the country and ordered two dozen or more dogs to be killed for no other reason than that the poor dogs were so unfortunate as to live in the neighborhood visited by the *suspected* dog. The day following the death of the mare, Mr. Garnett's dog and cat acted in a very suspicious manner ; in fact, *had fits !* Now, of course, the state of Mr. G.'s nervous system was in no condition to stand any such nonsense; he therefore took down his gun and shot them both. This was

* Virginia Medical Monthly, July, 1887.

still further proof, to those already convinced, that the mare was mad; but Mr. G. was one of the first to discover that some officious person had poisoned the dog and cat for fear they would go mad. Among the number of dogs ordered to be killed was one which disputed the right of way with the reprobate dog in question; a rough-and-tumble fight ensued, during which the reprobate dog literally "tore" the disputant dog, lacerated him, and laid him up for repairs. This dog now became "a suspect," but the owner refused to kill him. However, he was confined and closely watched, and has made a good recovery from his late indisposition, without any suspicious symptoms that I am aware of.

Running at the mare's side was her four-months-old colt, and the poor little colt also came in for its share of suspicion, and for weeks after was "a suspect." (I believe Mr. G. was advised to kill the colt.) The colt is now growing up to be a fine animal, and has never shown any symptons that indicated that universal specific for hydrophobia in the lower animals—the gun.

Now, when these animals were suspected and watched so closely, how much more carefully do you suppose Mr. G. was watched? Some of his best customers did not go to his store for some time, and some of those that did visit him were very careful not to excite him, while others felt safer when the counter was between them and Mr. Garnett.

We are happy to be able to state that the sensible procedures adopted by Dr. Harbaugh prevented a pseudo-lyssa in Mr. Garnett, who is doing well. Considerable excitement was created by the Haverstraw case. The diagnosis of rabies was confirmed by Dr. Wm. A. Hammond. It appears that the patient, while attempting to extract a foreign body from a pet dog's throat, was "nipped." His arm rapidly swelled up; intense pain followed. The physicians, according to the newspaper account, gave him chloroform and morphine, and in this condition he was judiciously informed that he would die in a short time. He died of blood-poisoning.

Another case is reported from Chicago. An editor of the chief medical monthly of that city made inquiry on our behalf and reports:

"The child snapped and howled during paroxysm, no evidence of hydrophobia in dog, nervous family, hydrophobia town talk for weeks before. The case was probably one of simple tetanus."

In view of the conflicting views as to the clinical character of rabies in man, we must insist that those who have opportunities for studying rabies in the dog, furnish us with better criteria than J. Wortley Axe in the *Veterinarian* of January, 1886. He states that if the stomach is void of food, and contains foreign matter, there is good ground for suspecting rabies. It is this same criterion which Mr. Victor Horsley used, and we are not surprised to find him, as well as the Hydrophobic Commission which entrusted its work to him, misled. No tyro in veterinary pathology but knows a dozen complaints in which the same condition is found. Nor are we satisfied with "the peculiar howl" which, on somewhat sounder grounds, is regarded as the characteristic peculiarity of a mad dog. Rost* says: "A dog which bites at all living objects and attacks his own master without provocation, is always to be regarded with suspicion and quarantined." This seems to us to besound, but it were well if we had some safe and reliable sign warning us of danger before the biting period comes on.

There is something mysterious about tetanic affections and other parasitic diseases of the nerve-centers, which may or may not bear on the possibility of a prolonged incubation of the various diseases which are confounded under the term rabies. Thus Cheesman† reports a case of traumatic tetanus, recovered from, and yet followed eight months later by tetanoid symptoms provoked by vaccination. We have ourselves, in a pure, uncomplicated case of epidemic cerebro-spinal meningitis, in a girl of thirteen, completely recovered from, seen a relapse of the opisthotomes, apparently brought on by the heat. All these and a host of similar facts indicate the necessity of renewed study of the entire subject. They point in this direction, that rabies is a collective name, indicating mental motor and sensory symptoms in the dog, which may be due to meningitis, insolation, acute delirium, typhoid fever, tetanus and perhaps an unknown disease. There is nothing surprising in the transmissibility by biting of some of these affections. But how rarely must this occur when there is not a single authentic case of a transmission of so-called rabies that cannot be accounted for either on the blood-poisoning or tetanus theory, in the whole domain of the United States, in two years?

After all the problems suggested in the foregoing are

---

* Quoted in *Fortschictte fur Medizin*, p. 783, 1886.
† Medical Record, May 8, 1886.

solved, it may occur to some one to examine into the question: What do the victims of M. Pasteur's method really die of? In the case of Dr. A. Stoboi, a Russian physician, who died after having been subjected to the Pasteur treatment, the dog that bit him being still alive,* the disease is pronounced as "dumb rabies." Similar symptoms were found in the other cases dying in England and France.† They closely resemble that "rabbit rabies" which M. Pasteur produced in his laboratory, and which Daremberg has imitated by using ordinary septic matter, thus repeating and confirming the results obtained by the writer.

With regard to the Pasteur institutes sporadically springing up in various parts of the world, they do not appear to be founded by those having the confidence of the best medical men in the community where they reside. A grave, ethical offense is charged in the *Virginia Medical Monthly* against the managers of the New York Pasteur Institute, which still has a nominal existence. A similar institution in Italy is denounced as a swindle‡ by Bordoni and Uffriduzzi, and has since vanished into the nothingness from which it sprang. More recently the New York Dog Pound was selected as the site of another Pasteur institute by an enthusiastic Hungarian chemist, but before the first inoculation could be carried out the agents of the Society for the Prevention of Cruelty to Animals interfered, and as the experimenter was unable to show any medical authority for his undertaking, he was cruelly choked off.

We expect at no distant date to present papers by two of our contributors, throwing light on certain experimental and clinical facts related to these questions.

---

DENTIGEROUS GROWTH.—We beg to call the attention of our readers to the case reported by Dr. A. H. Logan, in which he describes an operation performed by him on the horse, namely, the extirpation of a cystic tumor from the facial

* The Medical Standard, April, 1887.
† Paris Biological Society. British Medical Journal, p. 133, 1887, Vol. I
‡ Riforma medico, January 19, 1887.

sinus, which was found to contain 400 teeth; also to the article by Dr. J. Bland Sutton, on Teratomata, giving a clear description of the various tumors, in whose cysts are contained bodies so dissimilar in their nature.

These articles have mutual bearings. They show how the pathologist by his labors in the laboratory helps the practitioner in his practice, explaining to him the otherwise unaccountable phenomena which he encounters, whilst the latter, by reporting his case, affords the former food for thought and enables him to give practical demonstration of his scientific theories. C.

---

EQUINE SPINAL MENINGITIS.—It seems that this disease is now prevailing in New Jersey, and, as will be seen by the cases reported by Dr. Frey, it is evidently assuming the epidemic form in New York also. This is serious enough in itself, but how much more so when we consider the danger to human beings.

An authority who has made the subject a life-long study is of the opinion that we are on the eve of a great outbreak of this disease, similar to one fifteen years ago. He considers the principal cause to be dampness, filth and ill-ventilation, while a possible factor in its causation is bad food, such as mouldy grain, etc. He dwells on the importance of preventive measures—clean stables, well disinfected and the avoidance of manure pits.

It is then the duty of every Veterinarian to give this matter careful attention and to do all in his power to prevent the epidemic, or its extension if it should occur.

C.

NOTES.—We are pleased to inform our readers that Dr. R. S. Huidekoper has lately received the appointment of Honorary Foreign Associate of the Royal College of Veterinary Surgeons, London.

---

We have received the advanced slips of the proceedings of the ninth International Medical Congress, held at Washington September 5–10, through the courtesy of Messrs. William Wood & Company, Publishers of the

*Medical Record*, a weekly journal of medicine and surgery published in New York City.

---

We beg to call the attention of our readers to the paper by Prof. Walley on colic diseases. It is written as an introductory to another paper, which will be devoted to the most important subject connected with the colon, viz.: displacement, torsion, etc., a subject to which he has devoted much thought and many years of study, and but little understood by the great bulk of the members of the veterinary profession.

